# Seasonality of Common Human Coronaviruses, United States, 2014–2021^1^

**DOI:** 10.3201/eid2810.220396

**Published:** 2022-10

**Authors:** Melisa M. Shah, Amber Winn, Rebecca M. Dahl, Krista L. Kniss, Benjamin J. Silk, Marie E. Killerby

**Affiliations:** Centers for Disease Control and Prevention, Atlanta, Georgia, USA

**Keywords:** coronavirus, seasons, alphacoronavirus, betacoronavirus, seasonality, viruses, United States, COVID-19, 2019 novel coronavirus disease, coronavirus disease, severe acute respiratory syndrome coronavirus 2, SARS-CoV-2, viruses, respiratory infections, zoonoses

## Abstract

The 4 common types of human coronaviruses (HCoVs)—2 alpha (HCoV-NL63 and HCoV-229E) and 2 beta (HCoV-HKU1 and HCoV-OC43)—generally cause mild upper respiratory illness. Seasonal patterns and annual variation in predominant types of HCoVs are known, but parameters of expected seasonality have not been defined. We defined seasonality of HCoVs during July 2014–November 2021 in the United States by using a retrospective method applied to National Respiratory and Enteric Virus Surveillance System data. In the 6 HCoV seasons before 2020–21, season onsets occurred October 21–November 12, peaks January 6–February 13, and offsets April 18–June 27; most (>93%) HCoV detection was within the defined seasonal onsets and offsets. The 2020–21 HCoV season onset was 11 weeks later than in prior seasons, probably associated with COVID-19 mitigation efforts. Better definitions of HCoV seasonality can be used for clinical preparedness and for determining expected patterns of emerging coronaviruses.

The 4 common human coronaviruses (HCoVs), 2 alpha (HCoV-NL63 and HCoV-229E) and 2 beta (HCoV-HKU1 and HCoV-OC43) types, generally cause mild upper respiratory illness. The HCoVs are endemic among humans, as evidenced by sustained, widespread, continuous transmission, unlike the betacoronaviruses SARS-CoV (detected in 2002) and Middle East respiratory syndrome coronavirus (detected in 2012). An additional betacoronavirus, SARS-CoV-2, emerged in the human population in late 2019 and has become widespread. HCoVs circulate annually in the United States with a seasonal pattern, generally peaking during December–March and with the predominant types varying each year (*1*). 

Although HCoVs are known to have seasonal patterns, parameters of expected seasonality have not been defined. Given mitigation efforts and behavior changes resulting from the COVID-19 pandemic, national patterns of respiratory viruses, including influenza, differed during the 2020–21 season compared with previous seasons (*2*). Knowledge of changes to seasonal patterns in HCoV circulation is valuable for clinical and public health preparedness and may provide insight into transmission patterns for novel HCoVs. We analyzed circulation of 4 common HCoVs in the United States during July 2014–November 2021. 

## Methods

We analyzed circulation of HCoVs (HCoV-NL63, HCoV-229E, HCoV-HKU1, and HCoV-OC43, excluding SARS-CoV-2) by using data from the National Respiratory and Enteric Viruses Surveillance System (NREVSS, https://www.cdc.gov/surveillance/nrevss/labs/map.html), a passive surveillance system established by the Centers for Disease Control and Prevention (CDC) in the 1980s. NREVSS collects respiratory virus testing results from laboratories across the United States. Not all NREVSS laboratories submit results for all pathogen types, and ≈344 laboratories met the criteria for inclusion in our analysis. Clinical, public health, and commercial laboratories submit weekly aggregated numbers of tests performed and detections determined by reverse transcription PCR for the 4 common HCoV types. Although most NREVSS participants provide aggregated data, NREVSS also collects specimen-level data from a subset of 57 laboratories through the Public Health Laboratory Interoperability Project (PHLIP). Laboratories that submit data to NREVSS via PHLIP include information on patient age, administrative sex, and other respiratory virus test results.

The first week in the second surveillance year when common coronaviruses were surveyed in NREVSS ended on July 5, 2014. We included reports of specimens tested for HCoVs in NREVSS from the week ending July 5, 2014, through the week ending November 6, 2021. We excluded HCoV results without virus typing and data from laboratories that did not report any positive HCoV test results during the study period. We compiled total HCoV testing and positive detections by HCoV type, season, and US Census region. To characterize detections by patient age and sex, we used a subset of data submitted through PHLIP with specimens tested for all 4 HCoV types collected from June 29, 2014, through November 29, 2021 (because of data availability, we included a few additional weeks compared with NREVSS). We also examined codetection of HCoVs with other respiratory viruses in the PHLIP subset, including parainfluenza viruses, respiratory syncytial virus (RSV), human metapneumovirus, human adenovirus, rhinovirus/enterovirus, and influenza A and B viruses. We excluded specimens with panpositive results for codetections in the PHLIP subset.

We evaluated the onset and offset of seasons between MMWR week 31 (early August) through MMWR week 30 of the following year (https://ndc.services.cdc.gov/wp-content/uploads/MMWR_Week_overview.pdf) by using a method from NREVSS previously validated for RSV detection. This method (retrospective slope 10 method) is characterized by a centered, 5-week moving average of weekly detections with each seasonal peak normalized to 1,000 detections (*3*). We determined the absolute difference between normalized detections for each week and the previous week. We defined season onset as the second consecutive week with an absolute difference of >10 normalized detections and season offset as the last of 2 consecutive weeks when the number of normalized detections was greater than the number of normalized detections during the onset week. We determined seasonal characteristics nationally, including season onset, peak, and offset as well as season duration and percentage of annual detections that occurred within the season. We calculated the mean MMWR week for which seasonal inflections occurred by taking the mean of the MMWR weeks of the 6 seasons starting with 2014–15 and ending with 2019–20.

For our analyses, we used RStudio version 1.4.1106 (https://www.rstudio.com). This study was reviewed by CDC and conducted consistent with applicable federal law and CDC policy (45 C.F.R. part 46.102(l) (2), 21 C.F.R. part 56; 42 U.S.C. Sect. 241(d); 5 U.S.C. Sect. 552a; 44 U.S.C. Sect. 3501 et seq.).

## Results

**Figure 1:**
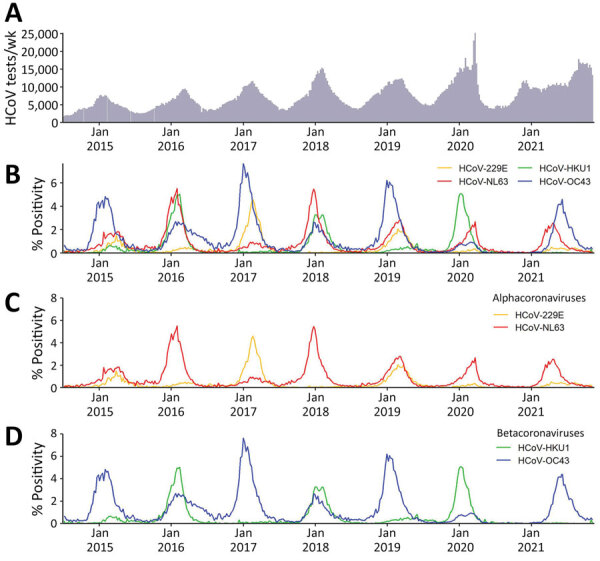
Total tests and percentage positivity of 4 common HCoVs from weekly aggregated data submitted to the National Respiratory and Enteric Virus Surveillance System, United States, July 2014–November 2021. A) Total specimens tested for all 4 HCoV types. B) Percentage positivity of the 4 HCoV types by week. C) Percentage positivity of the common alphacoronaviruses. D) Percentage positivity of the common betacoronaviruses. HCoVs, human coronaviruses.

We detected an HCoV of any type in 104,911 (3.6%) of 2,878,479 specimens with results submitted to NREVSS during the week ending July 5, 2014, through November 6, 2021. Among these 104,911 specimens, 40.1% were positive for HCoV-OC43, 27.8% for HCoV-NL63, 19.9% for HCoV-HKU1, and 12.2% for HCoV-229E. Weekly testing volumes were higher during March 2020, the onset of the COVID-19 pandemic, than during any other week in July 2014 and November 2021 (Figure 1, panel A). The predominant common HCoV type fluctuated by surveillance year (Figure 1, panel B).

**Figure 2:**
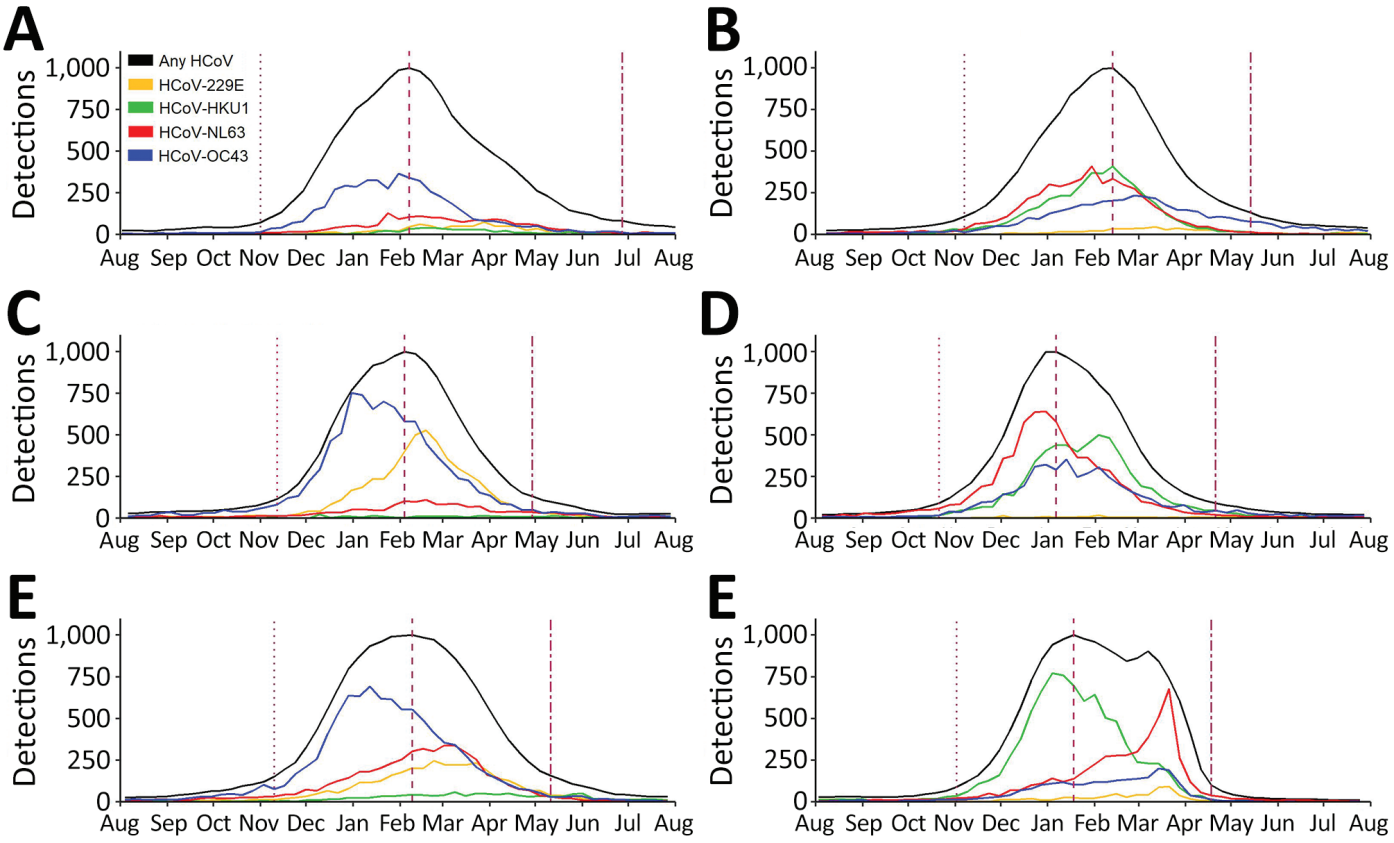
Total number of detections of the 4 common HCoVs, by week and season, from weekly aggregated data submitted to the National Respiratory and Enteric Virus Surveillance System, United States, July 2014–July 2020. The 3 vertical dotted lines, left to right, indicate the week of season onset, peak, and offset for all types combined (black line). These seasonal inflections were defined by using the retrospective slope 10 method, which uses a centered 5-week moving average of weekly detections with normalization to peak. The type-specific curves depict the actual number of detections; the black curve depicts specimens with any HCoV detections normalized to a peak of 1,000. HCoVs, human coronaviruses.

**Figure 3:**
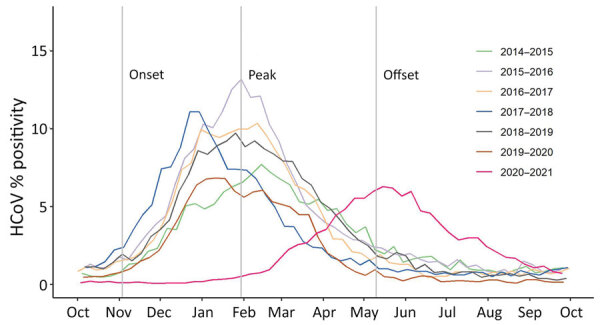
Percentage positivity and seasonal characteristics of common HCoVs, by season, from weekly aggregated data submitted to the National Respiratory and Enteric Virus Surveillance System, United States, October 2014–September 2021. Gray vertical lines indicate the mean starting week dates for season onset, peak, and offset for all seasons except 2020–21, based on the retrospective slope 10 method, which uses a centered 5-week moving average of weekly detections with normalization to peak to define seasonal inflections. The average onset week for the 6 seasons spanning 2014–2020 is MMWR week 44, average peak week is MMWR week 4, and the average offset week is MMWR week 19. For the 2020–21 season, the onset week is January 23 (MMWR week 3) and the peak week is May 22 (MMWR week 20) (not shown). HCoVs, human coronaviruses.

In the 6 HCoV seasons before the COVID-19 pandemic (i.e., excluding the 2020–21 season), seasonal onsets were during October–November, peaks during January–February, and seasonal offsets during April–June (Table 1; Figure 2). Specifically, the seasonal onset occurred on average during MMWR week 44 (range weeks 42–45), peak during week 4 (range weeks 1–6), and offset during week 19 (range weeks 17–25) (Table 1; Figure 3); 93.2% of all HCoV detections occurred between the onset and offset. The mean duration of the 6 seasons before the 2020–21 season was 25 weeks. The 2020–21 common HCoV season onset was delayed by 11 weeks compared with mean onset of prior seasons (Table 1; Figure 3). The number of days between onset and peak for the 2020–21 season (119 days) was longer than the mean observed for the prior 6 seasons (88 days). By November 2021, normalized values had not reached the requirement for offset for the 2020–21 season.

**Table 1:** Onset, peak, and offset dates for 4 common HCoVs and percentage detection, by season, from weekly aggregated data submitted to the National Respiratory and Enteric Virus Surveillance System, United States, July 2014–November 2021*

Season (MMWR wk)	Date (MMWR wk)				HCoV, %			
	Onset	Peak	Offset		OC43	NL63	HKU1	229E
2014–15 (31–30)	2014 Nov 1 (44)	2015 Feb 7 (5)	2015 Jun 27 (25)		6.3	23.5	58.8	11.4
2015–16 (31–30)	2015 Nov 7 (44)	2016 Feb 13 (6)	2016 May 14 (19)		31.1	34.8	29.4	4.8
2016–17 (31–30)	2016 Nov 12 (45)	2017 Feb 4 (5)	2017 Apr 29 (17)		1.8	10.0	56.8	31.4
2017–18 (31–30)	2017 Oct 21 (42)	2018 Jan 6 (1)	2018 Apr 21 (16)		33.5	39.3	25.8	1.4
2018–19 (31–30)	2018 Nov 10 (45)	2019 Feb 9 (6)	2019 May 11 (19)		5.8	25.3	49.9	19.0
2019–20 (31–30)	2019 Nov 2 (44)	2020 Jan 18 (3)	2020 Apr 18 (16)		51.9	29.5	14.2	4.3
2020–21 (31–44)	2021 Jan 23 (03)	2021 May 22 (20)	Not reached†		1.6	31.6	55.8	10.9

*Dates for season onset, peak, and offset for all seasons are based on the retrospective slope 10 method, which uses a centered 5-week moving average of weekly detections with normalization to peak to define seasonal inflections. Seasons were defined as starting on MMWR week 31 (beginning of August) of each year through MMWR week 30 (end of July) of the following year (https://ndc.services.cdc.gov/wp-content/uploads/MMWR_Week_ overview.pdf). For the last 2020–21 season, data were included from August 2021–November 2022 because of the delayed seasonal start. The percent of positive detections by each HCoV type by season (with the denominator being total positive detections of any HCoV type for that season) is included in the final 4 columns. HCoV, human coronavirus. †By November of 2021, normalized values had not reached the requirement for offset for the 2020–21 season.

The predominant type of alpha HCoV was HCoV-NL63 during 4 of the 7 seasons (Figure 1, panel C). When positivity for either HCoV-229E or HCoV-NL63 was >4%, positivity for the other alpha HCoV was <1% (Figure 1, panel C). The predominant beta HCoV type was HCoV-OC43, in a biennial pattern alternating with HCoV-HKU1 (Figure 1, panel D) except for 2017–18, when they were co-dominant. When positivity for either beta HCoV peaked at >4%, the other beta HCoV circulated at low levels (<1% positivity), except for the 2015–16 season (Figure 1, panel D). Across US Census regions, patterns of the predominant HCoV type were similar to national patterns (Appendix Figure 1).

Among 82,768 specimens from PHLIP tested for the 4 HCoVs from June 29, 2014, through November 29, 2021, any HCoV was detected in 5,204 (6.3%) (Table 2). We excluded 3 specimens because their reports indicated positive results for all respiratory viruses. Among 80,574 PHLIP specimens with information on patient sex, the percentage of specimens in which any HCoV was detected was similar among male (6.0%, 2,617/43,694) and female (6.2%, 2,284/36,880) patients. Of the 4 types of HCoV, detection of HCoV-OC43 was highest among children <1 years of age and adults 66–100 years of age (Table 2). HCoV-229E was detected in patients with the highest mean age and at the lowest percentages among children <5 years of age. Of specimens in which HCoV was detected, >1 type was detected in 64/5,204 (1.2%) and another respiratory virus was detected in 1,132/4,685 (24.2%); co-detection of influenza virus was most common (8.1%) (Table 3).

**Table 2:** Percentage positivity of the 4 common HCoVs, by patient sex and age categories, Public Health Laboratory Interoperability Project, United States, July 2014-November 2021

Patient category	HCoV, no. detected/no. tested (%)				
	Any	OC43	NL63	HKU1	229E
Total	5,204/82,768 (6.3)	2,056/82,768 (2.5)	1,519/82,768 (1.8)	962/8,276 (1.2)	732/82,768 (0.9)
Sex					
F	2,284/36,880 (6.2)	902/36,880 (2.4)	680/36,880 (1.8)	433/36,880 (1.2)	294/36,880 (0.8)
M	2,617/43,694 (6.0)	1,025/43,694 (2.3)	767/43,694 (1.8)	499/43,694 (1.1)	363/43,694 (0.8)
Age group, y*					
<1	578/4,788 (12.1)	271/4,788 (5.7)	144/4,788 (3.0)	110/4,788 (2.3)	62/4,788 (1.3)
1–5	795/9,501 (8.4)	349/9,501 (3.7)	271/9,501 (2.9)	146/9,501 (1.5)	47/9,501 (0.5)
6–17	517/9,568 (5.4)	147/9,568 (1.5)	207/9,568 (2.2)	92/9,568 (1.0)	77/9,568 (0.8)
18–65	2,738/50,240 (5.4)	994/50,240 (2.0)	781/50,240 (1.6)	566/50,240 (1.1)	425/50,240 (0.8)
>65	364/7,092 (5.1)	198/7,092 (2.8)	77/7,092 (1.1)	35/7,092 (0.5)	57/7,092 (0.8)

*Mean age (± SD), y: any, 25.8 (± 22.7); OC43, 27.3 (± 25.3); NL63, 22.9 (± 20.5); HKU1, 24.0 (± 19.8); 229E, 29.9 (± 21.8). HCoV, human coronavirus.

**Table 3:** Respiratory co-detections by common HCoV type, Public Health Laboratory Interoperability Project, July 2014–November 2021*

Virus co-detected	HCoV, no. tested/no. detected (%)				
	Any, n = 5,204	OC43, n = 2,056	NL63, n = 1,519	HKU1, n = 962	229E, n = 732
Any non-HCoV	1,132/4,685 (24.2)	447/1,831 (24.4)	289/1,357 (21.3)	217/893 (24.3)	161/657 (24.5)
Influenza virus	422/5,178 (8.1)	146/2,046 (7.1)	126/1,513 (8.3)	87/960 (9.1)	73/724 (10.1)
Rhinovirus/enterovirus	268/5,200 (5.2)	116/2,055 (5.6)	85/1,518 (5.6)	44/960 (4.6)	26/732 (3.6)
Respiratory syncytial virus	254/5,203 (4.9)	136/2,056 (6.6)	54/1,519 (3.6)	46/961 (4.8)	23/732 (3.1)
Respiratory adenovirus	158/5,202 (3.0)	80/2,054 (3.9)	38/1,519 (2.5)	28/962 (2.9)	16/732 (2.2)
Parainfluenza virus	99/5,204 (1.9)	43/2,056 (2.1)	19/1,519 (1.3)	19/962 (2.0)	20/732 (2.7)
Human metapneumovirus	95/5,204 (1.8)	29/2,056 (1.4)	27/1,519 (1.8)	26/962 (2.7)	15/732 (2.0)
HCoV co-detected					
>2 HCoV types	64/5,204 (1.2)	40/2,056 (1.9)	45/1,519 (3.0)	26/962 (2.7)	18/732 (2.5)
OC43	2,056/5,204 (39.5)	NA	21/1,519 (1.4)	6/962 (0.6)	14/732 (1.9)
NL63	1,519/5,204 (29.2)	21/2,056 (1.0)	NA	20/962 (2.1)	5/732 (0.7)
HKU1	962/5,204 (18.5)	6/2,056 (0.3)	20/1,519 (1.3)	NA	0/732 (0)
229E	732/5,204 (14.1)	14/2,056 (0.7)	5/1,519 (0.3)	0/962 (0)	NA

*HCoV, human coronavirus; NA, not applicable.

## Discussion

During the 6 seasons of HCoV circulation before the COVID-19 pandemic in 2020–21, the relative consistency of timing of seasonal onsets, peaks, and offsets indicates expected patterns in the seasonality of HCoVs in the United States. The predominant type of HCoV varied from season to season, but at least 1 alpha HCoV and 1 beta HCoV circulated each season, often in a biennial pattern, as in other northern latitude countries (*4*,*5*). This biennial pattern may reflect cross-immunity and waning population-level immunity to alpha and beta HCoVs from prior infections (*6*) because serologic and human studies suggest immunity to reinfection lasting ≈1 year (*7*,*8*).

Cross-reactive binding and neutralizing antibodies seem to be higher among common HCoV types within a genus (*9*). SARS-CoV-2 cross-reactive serum antibodies were present in serum before the COVID-19 pandemic, probably attributable to cross-immunity from prior HCoV infections, but they have not been shown to be protective against SARS-CoV-2 infection (*10*). Similarly, nonneutralizing antibodies to the common betacoronaviruses are boosted after SARS-CoV-2 infection (*10*), but potential effects of SARS-CoV-2 immunity on seasonal HCoV circulation remain unknown.

The seasonality of HCoVs probably results from a combination of viral, host, and environmental factors. In temperate climates, HCoVs circulate during the winter, aligned with cooler ambient temperature (*11*,*12*); seasonality is more varied and less predictable in tropical regions than in temperate regions (*4*,*13*). Colder temperatures are thought to improve the stability of enveloped viruses (*14*). In addition, lower temperatures lead to drying of airways and can increase host susceptibility to infection. Environmental factors can also lead to behavior change, which affects the spread of HCoVs, such as from more indoor human contact during winter (*14*). Similarly, other widespread behavior changes could alter the seasonality of HCoV circulation.

The pattern of HCoV circulation during the 2020–21 season differed from that during prior seasons; onset was delayed by 11 weeks compared with the mean of prior seasons, and duration to peak was extended. The 2020–21 season offset could not be determined because the number of detections had not fallen to low enough levels at the time of this analysis. In the United States, the seasonal starts of RSV and parainfluenza virus circulation were delayed during 2020–21, and influenza virus and human metapneumovirus circulation was attenuated (*2*,*15*). Activity of rhinovirus/enterovirus and human adenovirus was lower than usual at the beginning of the season, but activity increased to prepandemic levels later in the season. These changes are probably attributable in part to implementation of COVID-19 pandemic mitigation measures, such as decreased domestic and global travel, use of face masks, school and office closures, and physical distancing (*2*).

Certain clinical and phenotypic differences in the 4 seasonal HCOVs have been observed (e.g., distribution of patient sex and age and virus pathogenicity). HCoV-OC43 has been reported as the most prevalent of the 4 common HCoVs, consistent with our findings (*12*). A previous study reported male sex as being associated with higher odds of HCoV positivity (*16*), but in our study, likelihood of HCoV detection was not higher among male patients.

HCoVs circulate seasonally with other respiratory viruses, including RSV, influenza virus, and rhinoviruses; co-infections are not uncommon (*17*,*18*). We similarly show high levels of HCoV co-detections (24%), particularly with influenza virus, which is probably an underestimate because only influenza virus detections from respiratory panels are included in the PHLIP dataset. Further work is needed to understand mechanisms of viral interference and the role of virus co-infections in the pathophysiology of illness and circulation of respiratory viruses.

Among the limitations of this investigation, testing patterns for respiratory viruses changed during the 2020–21 season because of delayed routine healthcare and an emphasis on SARS-CoV-2 testing, which affects comparison with earlier seasons. The representativeness of co-detections reported (i.e., true burden of illness) could not be evaluated because this PHLIP platform does not include reasons for testing (e.g., symptomatic disease); positive detections may be more likely to be reported than negative detections. The NREVSS platform represents a geographically heterogenous subset of all US laboratories but may not be nationally or locally representative. Furthermore, types of laboratory participation and the process for obtaining the subset of specimen level data for PHLIP are not fully comparable with the overall NREVSS platform.

According to our analysis, a typical common HCoV season in the United States generally starts during October–November, peaks near the end of January, and ends during April–June. This knowledge of expected seasonal variation in HCoV circulation is useful for public health preparedness and clinical management of patients. Clinicians and the public health community should be aware that patterns of HCoV circulation changed during 2020–21 and that trends in future seasons may also deviate from trends before the COVID-19 pandemic.

Appendix Supplementary information for study of seasonality of common human coronaviruses, United States, 2014–2021.

## References

[1] Killerby ME, Biggs HM, Haynes A, Dahl RM, Mustaquim D, Gerber SI, et al. Human coronavirus circulation in the United States 2014-2017. J Clin Virol. 2018;101:52–6. 10.1016/j.jcv.2018.01.019 29427907 PMC7106380

[2] Olsen SJ, Winn AK, Budd AP, Prill MM, Steel J, Midgley CM, et al. Changes in influenza and other respiratory virus activity during the COVID-19 pandemic—United States, 2020–2021. MMWR Morb Mortal Wkly Rep. 2021;70:1013–9. 10.15585/mmwr.mm7029a1 34292924 PMC8297694

[3] Midgley CM, Haynes AK, Baumgardner JL, Chommanard C, Demas SW, Prill MM, et al. Determining the seasonality of respiratory syncytial virus in the United States: the impact of increased molecular testing. J Infect Dis. 2017;216:345–55. 10.1093/infdis/jix275 28859428 PMC5712458

[4] Li Y, Wang X, Nair H. Global seasonality of human seasonal coronaviruses: a clue for postpandemic circulating season of severe acute respiratory syndrome coronavirus 2? J Infect Dis. 2020;222:1090–7. 10.1093/infdis/jiaa436 32691843 PMC7454715

[5] Hawkes MT, Lee BE, Kanji JN, Zelyas N, Wong K, Barton M, et al. Seasonality of respiratory viruses at northern latitudes. JAMA Netw Open. 2021;4:e2124650. 10.1001/jamanetworkopen.2021.24650 34529066 PMC8446819

[6] Kissler SM, Tedijanto C, Goldstein E, Grad YH, Lipsitch M. Projecting the transmission dynamics of SARS-CoV-2 through the postpandemic period. Science. 2020;368:860–8. 10.1126/science.abb5793 32291278 PMC7164482

[7] Edridge AWD, Kaczorowska J, Hoste ACR, Bakker M, Klein M, Loens K, et al. Seasonal coronavirus protective immunity is short-lasting. Nat Med. 2020;26:1691–3. 10.1038/s41591-020-1083-1 32929268

[8] Callow KA, Parry HF, Sergeant M, Tyrrell DA. The time course of the immune response to experimental coronavirus infection of man. Epidemiol Infect. 1990;105:435–46. 10.1017/S0950268800048019 2170159 PMC2271881

[9] Gorse GJ, Donovan MM, Patel GB. Antibodies to coronaviruses are higher in older compared with younger adults and binding antibodies are more sensitive than neutralizing antibodies in identifying coronavirus-associated illnesses. J Med Virol. 2020;92:512–7. 10.1002/jmv.25715 32073157 PMC7166442

[10] Anderson EM, Goodwin EC, Verma A, Arevalo CP, Bolton MJ, Weirick ME, et al.; UPenn COVID Processing Unit. Seasonal human coronavirus antibodies are boosted upon SARS-CoV-2 infection but not associated with protection. Cell. 2021;184:1858–1864.e10. 10.1016/j.cell.2021.02.010 33631096 PMC7871851

[11] Aldridge RW, Lewer D, Beale S, Johnson AM, Zambon M, Hayward AC, et al.; Flu Watch Group. Seasonality and immunity to laboratory-confirmed seasonal coronaviruses (HCoV-NL63, HCoV-OC43, and HCoV-229E): results from the Flu Watch cohort study. Wellcome Open Res. 2020;5:52. 10.12688/wellcomeopenres.15812.2 33447664 PMC7786426

[12] Nickbakhsh S, Ho A, Marques DFP, McMenamin J, Gunson RN, Murcia PR. Epidemiology of seasonal coronaviruses: establishing the context for the emergence of coronavirus disease 2019. J Infect Dis. 2020;222:17–25. 10.1093/infdis/jiaa185 32296837 PMC7184404

[13] Komabayashi K, Seto J, Matoba Y, Aoki Y, Tanaka S, Ikeda T, et al. Seasonality of human coronavirus OC43, NL63, HKU1, and 229E infection in Yamagata, Japan, 2010-2019. Jpn J Infect Dis. 2020;73:394–7. 10.7883/yoken.JJID.2020.525 32741934

[14] Price RHM, Graham C, Ramalingam S. Association between viral seasonality and meteorological factors. Sci Rep. 2019;9:929. 10.1038/s41598-018-37481-y 30700747 PMC6353886

[15] Olsen SJ, Azziz-Baumgartner E, Budd AP, Brammer L, Sullivan S, Pineda RF, et al. Decreased influenza activity during the COVID-19 pandemic–United States, Australia, Chile, and South Africa, 2020. MMWR Morb Mortal Wkly Rep. 2020;69:1305–9. 10.15585/mmwr.mm6937a6 32941415 PMC7498167

[16] Dyrdak R, Hodcroft EB, Wahlund M, Neher RA, Albert J. Interactions between seasonal human coronaviruses and implications for the SARS-CoV-2 pandemic: A retrospective study in Stockholm, Sweden, 2009-2020. J Clin Virol. 2021;136:104754. 10.1016/j.jcv.2021.104754 33601153 PMC7869750

[17] Gaunt ER, Hardie A, Claas EC, Simmonds P, Templeton KE. Epidemiology and clinical presentations of the four human coronaviruses 229E, HKU1, NL63, and OC43 detected over 3 years using a novel multiplex real-time PCR method. J Clin Microbiol. 2010;48:2940–7. 10.1128/JCM.00636-10 20554810 PMC2916580

[18] Lu R, Yu X, Wang W, Duan X, Zhang L, Zhou W, et al. Characterization of human coronavirus etiology in Chinese adults with acute upper respiratory tract infection by real-time RT-PCR assays. PLoS One. 2012;7:e38638. 10.1371/journal.pone.0038638 22719912 PMC3376151

