# Supplementary material for: Seasonality of Common Human Coronaviruses, United States, 2014–2021
Source: Emerg Infect Dis. 2022 Oct;28(10):1970–6. doi: 10.3201/eid2810.220396 (PMC9514339; doi:10.3201/eid2810.220396)
Supplement: Appendix — Supplementary information for study of seasonality of common human coronaviruses, United States, 2014–2021. [file 22-0396-Techapp-s1.pdf]

Article DOI: https://doi.org/10.3201/eid2810.220396

Seasonality of Common Human Coronaviruses, United States, 2014–2021

Appendix

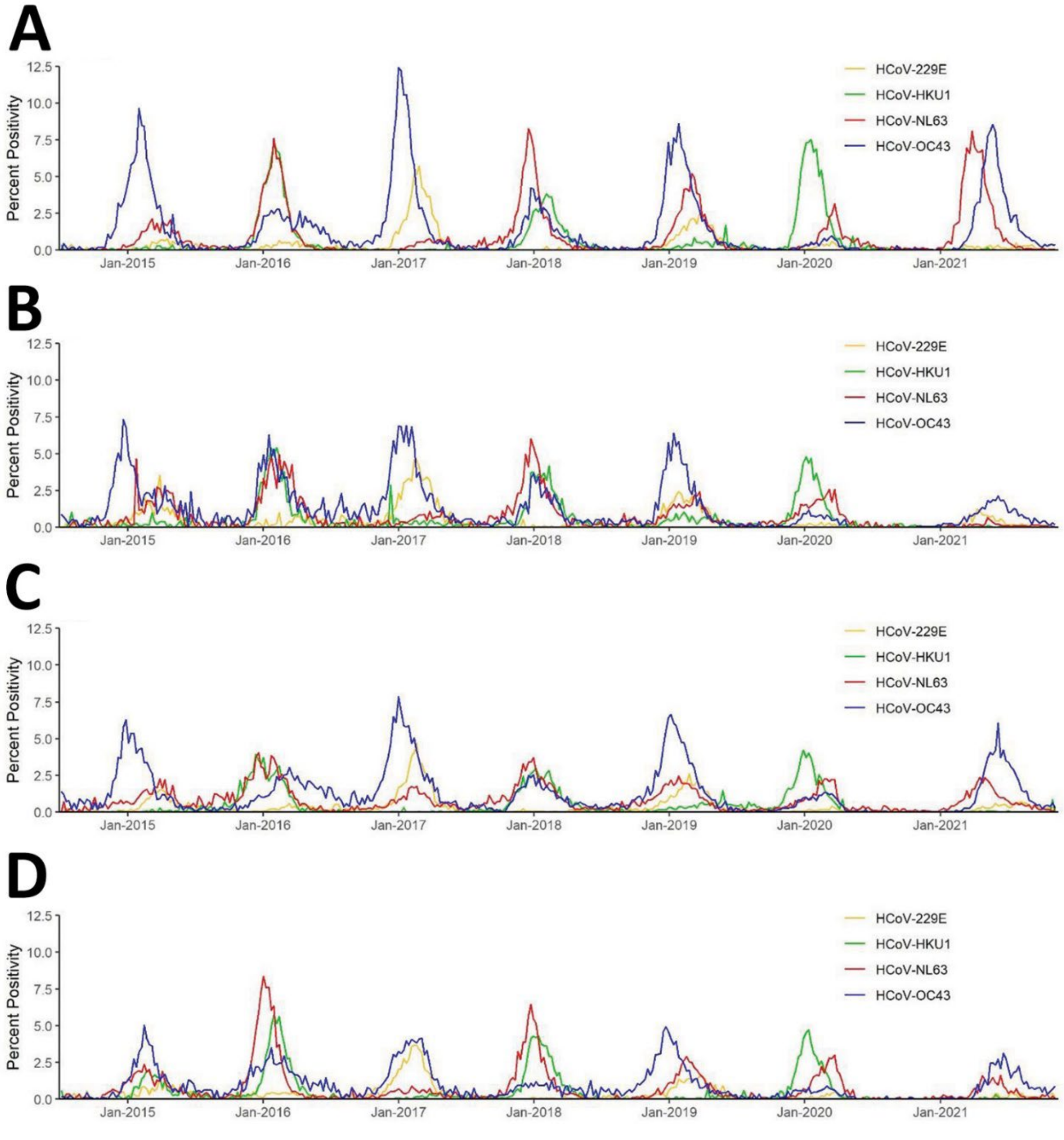

Appendix Figure. Percent positivity of the four common human coronaviruses (HCoVs) including HCoV-229E, HCoV-HKU1, HCoV-NL63, and HCoV-OC43 by week and United States Census region (Midwest, A; Northeast, B; South, C; and West, D) from weekly aggregated data submitted to the National Respiratory and Enteric Virus Surveillance System (NREVSS), July 2014–November 2021.
